# Intracellular Polyphosphate Levels in *Gluconacetobacter diazotrophicus* Affect Tolerance to Abiotic Stressors and Biofilm Formation

**DOI:** 10.1264/jsme2.ME18044

**Published:** 2018-11-07

**Authors:** Mariana Grillo-Puertas, Paola Delaporte-Quintana, Raúl Osvaldo Pedraza, Viviana Andrea Rapisarda

**Affiliations:** 1 Instituto Superior de Investigaciones Biológicas (INSIBIO), CONICET-UNT, and Instituto de Química Biológica, “Dr. Bernabé Bloj”, Facultad de Bioquímica, Química y Farmacia UNT. San Miguel de Tucumán, Tucumán Argentina; 2 Facultad de Agronomía y Zootecnia UNT San Miguel de Tucumán, Tucumán Argentina

**Keywords:** *Gluconacetobacter diazotrophicus*, plant growth-promoting bacteria, polyphosphate, phosphate, stress tolerance, biofilm formation

## Abstract

*Gluconacetobacter diazotrophicus* is a plant growth-promoting bacterium that is used as a bioinoculant. Phosphate (Pi) modulates intracellular polyphosphate (polyP) levels in *Escherichia coli*, affecting cellular fitness and biofilm formation capacity. It currently remains unclear whether environmental Pi modulates polyP levels in *G. diazotrophicus* to enhance fitness in view of its technological applications. In high Pi media, cells accumulated polyP and degraded it, thereby improving survival, tolerance to environmental stressors, biofilm formation capacity on abiotic and biotic surfaces, and competence as a growth promoter of strawberry plants. The present results support the importance of Pi and intracellular polyP as signals involved in the survival of *G. diazotrophicus*.

Polyphosphate (polyP) is a linear polymer of inorganic phosphate (Pi) residues linked by phosphoanhydride bonds and is present in bacteria, archaea, fungi, protozoa, plants, and animals (19). PolyP is involved in physiological and regulatory mechanisms, including motility, competence, biofilm formation, and virulence, in several bacteria and parasites (10, 12, 13, 21, 29). It also plays roles in bacterial survival in the stationary phase and responses to nutrient starvation and other stress conditions (7, 11, 29, 32). In the *Escherichia coli* K-12 strain, polyP accumulates during the exponential phase and is degraded in the early stationary phase under Pi limitation or sufficiency (23, 32). However, in media containing high Pi concentrations (>25–37 mM), cells maintain the intracellular polyP pool during the stationary phase (10, 31). PolyP degradation in Pi sufficiency triggers biofilm formation via the LuxS quorum sensing system (10). On the other hand, when polymer levels were maintained, the addition of copper to cells was shown to induce its degradation with the consequential detoxification of metal ions (11).

Plant growth-promoting bacteria (PGPB) represent a promising alternative to agrochemicals because they may be used as bioinoculants (3). However, environmental conditions, such as humidity, temperature, salinity, heavy metals, pesticides, and plant-related compounds, influence the effectiveness of PGPB before, during, and after the inoculation of plants (22). Among PGPB, *Gluconacetobacter diazotrophicus*, a N_2_-fixing endophyte that was initially isolated from sugarcane (27, 30), has been attracting increasing attention. The use of *G. diazotrophicus* as a bioinoculant still requires studies on its physiological properties, including how this bacterium responds to extracellular compounds. Accordingly, the aim of the present study was to analyze the effects of medium Pi concentrations on the modulation of intracellular polyP levels in *G. diazotrophicus*, which may affect biofilm formation and survival to the stressors typically present in its natural environment.

Growth curves of *G. diazotrophicus* in media containing different Pi concentrations were carried out (Fig. 1). PAL5 strain (ATCC 49037) cells were previously grown for 48 h in LGIP medium (containing 6 mM Pi) pH 5 (4) and incubated in media with different Pi concentrations (LGIP1, LGIP3, LGIP, LGIP10, LGIP20, LGIP30, and LGIP40 containing 1, 3, 6, 10, 20, 30 and 40 mM Pi, respectively). Bacterial growth was followed under shaken conditions at 30°C for 120 h by measuring A_560nm_. Specific growth rates (μ) were calculated from five consecutive A_560_ measurements in the exponential phase (μ=Δln A_560_/Δt, where t is time) (5). Cell growth improved when medium Pi concentrations increased from 1 to 30–40 mM, with μ values of 0.067 and 0.132 min^−1^, respectively. Based on the differential growth profiles obtained, LGIP1, LGIP10, and LGIP30 media were selected for subsequent experiments.

The ability of PAL5 cells to tolerate different external agents (NaCl, H_2_O_2_, and copper-related microbicides) was evaluated in the selected media (Fig. 2A). Cells grown in LGIP1 were sensitive to 200 mM NaCl, 100 ppm CuSO_4_, 50 ppm Cu(OH)_2_, and 50 ppm Cu_2_O, whereas cells grown in LGIP30 were tolerant. Cells in LGIP10 were tolerant to 200 mM NaCl and 100 ppm CuSO_4_, but were unable to grow in 50 ppm Cu(OH)_2_ or Cu_2_O. PAL5 cells exhibited intrinsic tolerance to H_2_O_2_ that was not significantly enhanced in high Pi medium (data not shown). Copper resistance in high Pi media was previously demonstrated in other microorganisms, such as *E. coli* and *Anabaena variabilis* (11, 14). Copper salts are used as antimicrobial agents in crop protection against several diseases and are currently accumulating in soils, becoming toxic to plants and microorganisms (24). Soil salinization is also a serious stress condition, affecting crop productivity as well as microbial activity in the rhizosphere, which further influences plant growth (33). In this context, the ability of *G. diazotrophicus* PAL5 to tolerate copper compounds and salinity is relevant in view of its biotechnological applications.

PAL5 biofilm formation in the selected media was tested on glass or polystyrene surfaces (Fig. 2B). The capacity to form biofilms on both surfaces was enhanced with the increase in the Pi concentrations, and was approximately three-fold higher in LGIP30 than in LGIP1. The differential amounts of biofilms among several Pi concentrations were not due to differences in cell density because planktonic cells were similar in both media (A_560nm_ 0.189±0.015). The adhesion of PAL5 to strawberry roots in Hoagland medium (9) containing 1 or 30 mM Pi was quantified by crystal violet staining (Fig. 2C). Biofilm formation g^−1^ of strawberry root was significantly higher in 30 mM Pi than in 1 mM Pi according to abiotic surface data. Bacterial adhesion on roots was also analyzed by SEM (Fig. 2D). In 1 mM Pi, isolated bacteria adhered to surfaces covered by root components, while a robust biofilm with an abundant extracellular matrix formed on roots in 30 mM Pi. Planktonic cells from the strawberry root assays were similar under all conditions tested (~3–4 10^7^ UFC mL^−1^). Delaporte-Quintana *et al.* (8) recently reported the capacity of the PAL5 strain to promote strawberry plant growth, demonstrating a plant-bacterium interaction. In the present study, biofilm formation with 30 mM Pi is important considering that root colonization is required for the diverse beneficial effects of PGPB (20, 25). Thus, the capacity of *G. diazotrophicus* to improve strawberry plant growth under different Pi conditions was evaluated as follows. Plant materials and substrates were prepared according to Delaporte-Quintana *et al.* (8). The following treatments were then applied: i) plants receiving Hoagland nutrient solution without Pi (P0); ii) plants receiving the nutrient solution including 1 mM soluble potassium phosphate (P1); iii) plants receiving the nutrient solution including 1 mM soluble potassium phosphate and inoculated by immersion for 30 min in the *G. diazotrophicus* PAL5 (~10^8^) suspension (P1+PAL5); iv) plants receiving the nutrient solution including 30 mM soluble potassium phosphate (P30); v) plants receiving the nutrient solution including 30 mM soluble potassium phosphate and inoculated by immersion for 30 min in the *G. diazotrophicus* PAL5 (~10^8^) suspension (P30+PAL5). Plants were maintained in the growing chamber (8) for 26 d from the beginning of the assay. During this period, plants received distilled water when necessary and 5 mL of Hoagland nutrient solution modified according to the applied treatments once a week. The growth index (GI) was assessed based on the total biomass dry weight at the beginning and the end of the experiment in each treatment. The highest GI value was observed when the plant was exposed to 30 mM in the presence of PAL5 (P30+PAL5) and the lowest when Pi was not included in the nutrient solution (P0) (Fig. 3, upper panel). Intermediate GI values were obtained under the other assay conditions. Root area was assessed at the end of the experiment using the gravimetric method described by Carley and Watson (6). Values corresponding to the P0, P1, and P1+PAL5 conditions were lower than those for the P30 and P30+PAL5 conditions (Fig. 3, bottom panel). The Most Probable Number (MPN) of diazotrophs associated with root samples was evaluated at the end of the assay using the McCrady Table for 3 repetitions according to Pedraza *et al.* (28). MPN corresponded to 1.5×10^4^ and 4.5×10^4^ diazotrophs g^−1^ of fresh root under the P1+PAL5 and P30+PAL5 conditions, respectively, while no diazotroph was found in non-inoculated plants. These results indicate that even when Pi was easily available to plants in P1 and P30, the addition of *G. diazotrophicus* exerted beneficial effects on strawberry plant growth, mostly under the 30 mM condition. This fact is related to the high amount of diazotrophs that associated with the roots under the 30 mM condition, which is consistent with differential adhesion on biotic and abiotic surfaces described above.

PolyP was measured in *G. diazotrophicus* PAL5 to establish whether Pi concentrations in culture media influence intracellular polymer levels (Fig. 4A). In LGIP1 medium, polyP levels remained low throughout the growth curve, while polyP accumulated up to 48–72 h in LGIP10 and LGIP30 media and degraded thereafter. Cells grown in LGIP10 had lower polyP levels in the stationary phase than those in LGIP30. According to these results, high intracellular polyP levels in bacteria grown in high Pi media correlated with stress tolerance under these conditions. The response of polyP to environmental Pi was previously reported in *E. coli* K12, uropathogenic *E. coli* (UPEC), and *Lactobacillus rhamnosus* (7, 12, 32). However, the modulation of polymer levels differed with the microorganism used. According to the three UPEC isolates studied and the non-pathogenic laboratory strain (12, 32), Pi-dependent polyP fluctuations in *G. diazotrophicus* were only similar to those in MGP45 UPEC. *G. diazotrophicus* PAL5 biofilm formation on abiotic surfaces was achieved when the bacterium was able to accumulate polyP and subsequently degrade it. This is consistent with previous findings related to biofilm formation by pathogenic and non-pathogenic *E. coli* (10, 12). Thus, the degradation of preformed polyP is a common feature related to metabolic responses, such as stress tolerance and biofilm formation. Bacterial exopolysaccharides participate in adherence to and the colonization of surfaces (18, 34). Idogawa *et al.* (15) showed that the production of the exopolysaccharide levan by *G. diazotrophicus* PAL5 was enhanced in medium with a high Pi concentration, denoted by a strong mucous phenotype. Furthermore, the involvement of levan in biofilm formation and tolerance to desiccation, osmotic pressure, and NaCl stress has been demonstrated in *G. diazotrophicus* (34). In our experimental approach, colonies in LGIP30 showed a mucous aspect (not shown), suggesting the production of extracellular compounds under this condition. Fluctuations in polyP levels may influence their production, as described for *Pseudomonas aeruginosa*, in which the synthesis of exopolysaccharides is regulated by polyP levels (17).

An important function of polyP is the regulation of heavy metal homoeostasis, which contributes to their detoxification (16). To obtain insights into the involvement of polyP in *G. diazotrophicus* tolerance to external stressors, the effects of a 1-h incubation with copper on polyP levels in the stationary phase were evaluated (Fig. 4B). The addition of copper induced polyP degradation when cells had elevated polymer levels in the stationary phase (72 h LGIP10 or LGIP30 conditions). These cells were tolerant to metal exposure, while cells with low polyP values (LGIP1 and 120 h LGIP10 conditions) were sensitive to copper. Cells grown in LGIP30 for 120 h partially tolerated copper, indicating that the intermediate polyP level under this condition was sufficient to counteract the deleterious effects of the metal. Collectively, these data demonstrated that PAL5 copper tolerance correlated with the degradation of preformed polyP. In agreement with these results, polyP degradation was identified as a step in an alternative mechanism to detoxify metals in *E. coli* (13), acidophilic bacteria (1), and archaea (31). Further studies are needed to elucidate other key factors involved in the polyP-dependent metal detoxification system in *G. diazotrophicus*.

The present results demonstrated that Pi concentrations in culture media modulate intracellular polyP levels in *G. diazotrophicus*, linking the polymer with the capacity of this bacterium to improve survival, overcome environmental stress, and form biofilms. These metabolic abilities enhance PAL5 strain competence as a promoter of strawberry plant growth. Our findings constitute a significant contribution towards clarifying the importance of Pi and intracellular polyP as signals involved in metabolic adaptations that favor *G. diazotrophicus* capacities as a bioinoculant for agricultural purposes.

## Figures and Tables

**Fig. 1:**
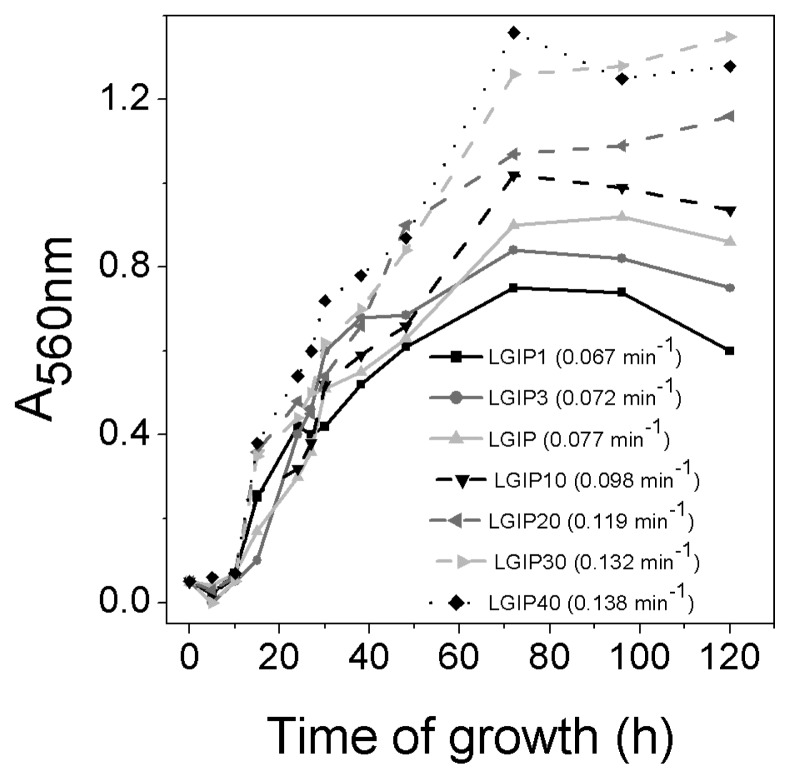
Growth curves of *Gluconacetobacter diazotrophicus* in media with different Pi concentrations. PAL5 strain cells were previously grown for 48 h in LGIP pH 5 medium and diluted in glass flasks containing media with different Pi concentrations (LGIP1, LGIP3, LGIP, LGIP10, LGIP20, LGIP30, and LGIP40, containing 1, 3, 6, 10, 20, 30, and 40 mM Pi, respectively). Bacterial growth was followed at 30°C for 120 h under shaken conditions by measuring A_560nm_. Specific growth rate (μ) values are shown in parentheses. Data are representative of at least six independent experiments.

**Fig. 2:**
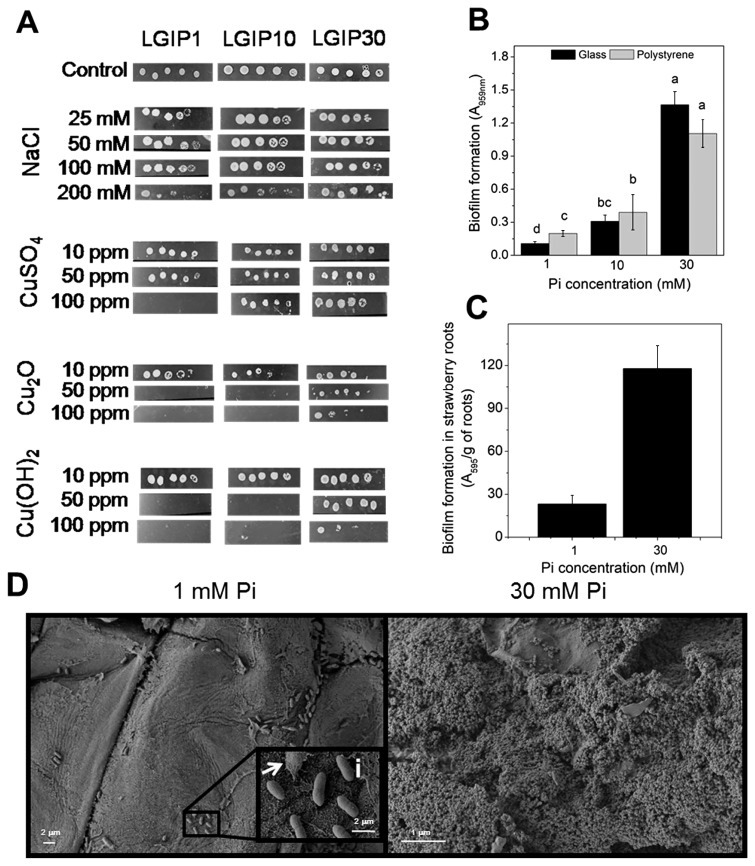
Tolerance to abiotic agents and biofilm formation capacity of *Gluconacetobacter diazotrophicus*. **A.** PAL5 cells were grown in LGIP medium at 30°C and 150 rpm for 48 h, harvested, and resuspended in fresh medium to A_560nm_=1 (~5×10^8^ CFU mL^−1^). Tolerance to NaCl and copper-related microbicides was evaluated by spotting a 1/10 serial dilution cell suspension on LGIP1, LGIP10, or LGIP30-agar plates supplemented or not with the indicated concentrations of the compounds. Plates were incubated at 30°C for 48 h. Data are representative of at least six independent experiments. **B.** ON cultures in LGIP were diluted to A_560nm_=0.1 with fresh LGIP1, LGIP10, or LGIP30 medium and incubated under static conditions at 30°C for 48 h in microtiter plates or glass tubes. The quantification of attached cells was performed by measuring A_595nm_, according to O’Toole and Kolter (26). Data represent the mean±SD of at least 4 independent experiments. Different letters indicate significant differences according to Tukey’s test with a *P*-value of 0.05. **C** and **D.** The adhesion of PAL5 cells to the roots of strawberry plants in 1 or 30 mM Pi solution was quantified by the O’Toole and Kolter technique (**C**) and visualized by SEM (**D**). Briefly, axenic *in vitro* plants of strawberry (*Fragaria ananassa*, Duch) cv. ‘Pájaro’ were transferred to glass bottles with Hoagland solution containing 1 or 30 mM Pi. Plants were inoculated with 1 mL of bacterial suspension (~10^6^ CFU mL^−1^) previously grown in LGIP medium for 48 h and were placed in a growth chamber at 25°C with a 16-h photoperiod and 60% relative humidity. After 72 h, root samples were collected and rinsed with distilled water. SEM images were obtained using a Carl Zeiss SUPRA-55 scanning electron microscope (CIME, Tucumán-Argentina). Data and images are representative of 2 independent experiments, consisting of three plants under each condition and three root samples for each plant. Magnification in (**D**) panels: 5,000×; inset i: 50,000×.

**Fig. 3:**
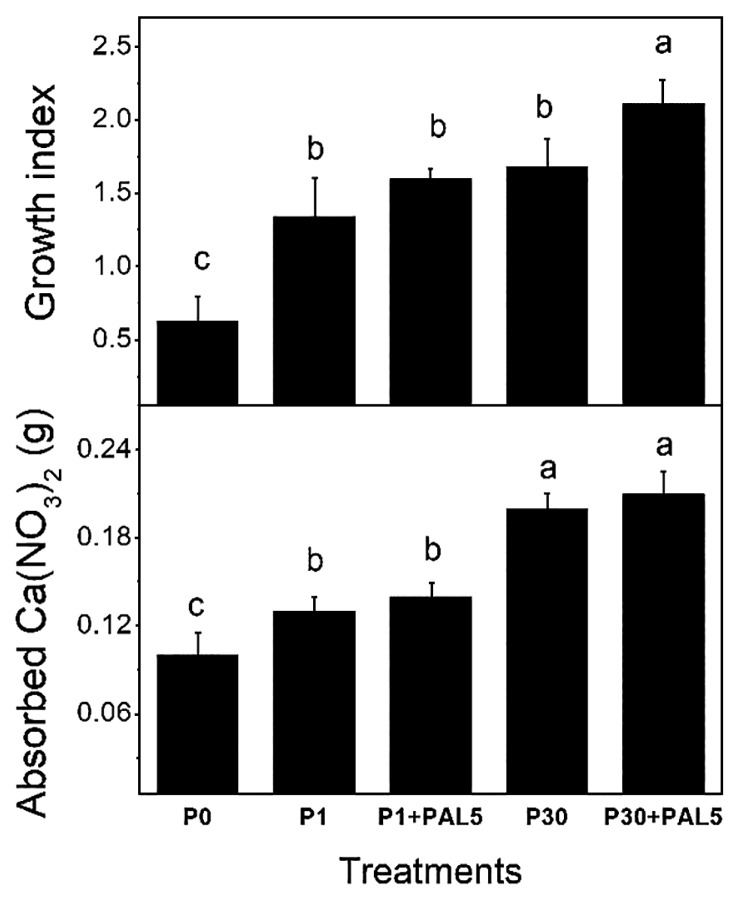
Growth promotion of strawberry plants. The plant growth index (upper panel) was assessed in consideration of total biomass dry weight changes after 26 d of treatment. Plants were removed from the pots and the substrate was carefully detached. Roots were washed with sterile water and dried using tissue paper. Plants were then dried in an oven at 60°C until a constant weight was reached. The root area (bottom panel) was expressed as g of Ca(NO_3_)_2_ absorbed by the roots. Data represent the mean±SD of two independent experiments, with three replicates for each condition. Similar letters did not differ significantly according to Tukey’s test with a *P*-value of 0.05. Applied treatments: **P0**, Hoagland nutrient solution without Pi; **P1**, nutrient solution including 1 mM soluble potassium phosphate; **P1+PAL5**, nutrient solution including 1 mM soluble potassium phosphate with the *G. diazotrophicus* PAL5 suspension; **P30**, nutrient solution including 30 mM soluble potassium phosphate; **P30+PAL5**, nutrient solution including 30 mM soluble potassium phosphate with the *G. diazotrophicus* PAL5 suspension.

**Fig. 4:**
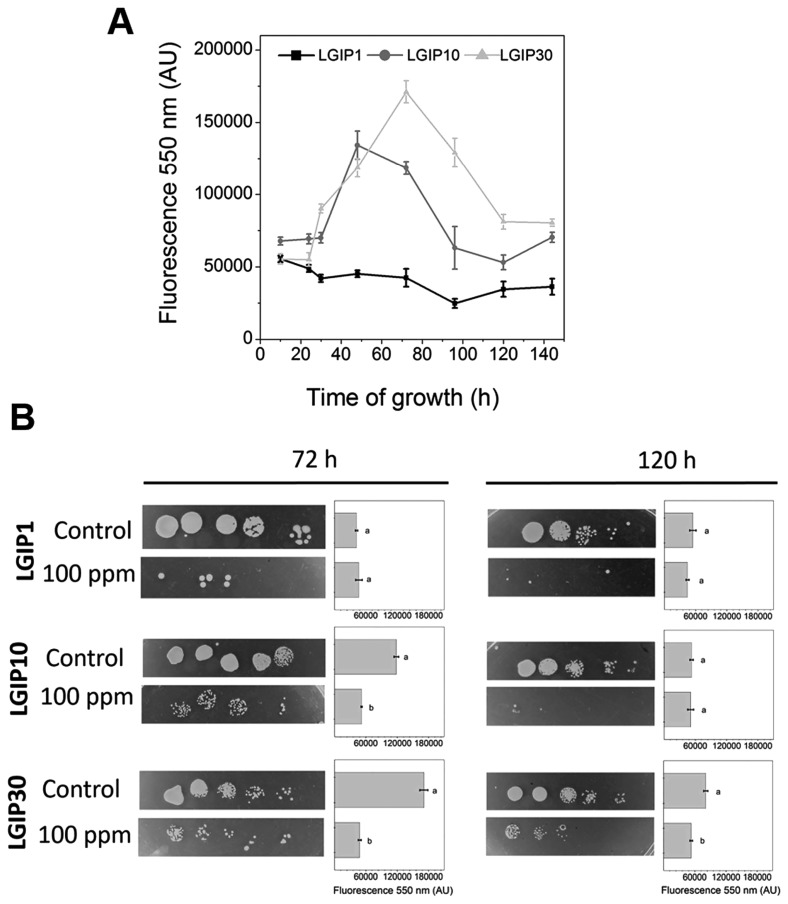
Polyphosphate in *Gluconacetobacter diazotrophicus* and the effect of copper on polymer levels in the stationary phase. **A.** PAL5 cells were grown at 30°C for 140 h in the indicated media. At different times, intracellular polyP levels were quantified by the emission fluorescence of the polyP-DAPI (4′,6-diamidino-2-phenylindole) complex at 550 nm (2, 10, 32). **B.** Copper tolerance (plate photographs) and polyP levels (bar graphs) were evaluated in a short-time metal exposition assay. PAL5 cells were grown in the indicated liquid media for 72 or 120 h, harvested by centrifugation, resuspended in fresh media to an A_560nm_=1, and incubated with shaking at 30°C for 1 h without or with 100 ppm CuSO_4_. Metal tolerance was evaluated by spotting 1/10 serial dilutions on LGIP-agar and incubating at 30°C for 48 h. Fluorescence is expressed in AU as the average±SD of four independent experiments. Under each condition, different letters indicate significant differences according to Tukey’s test with a *P*-value of 0.05.
